# Plastoquinone and Ubiquinone in Plants: Biosynthesis, Physiological Function and Metabolic Engineering

**DOI:** 10.3389/fpls.2016.01898

**Published:** 2016-12-16

**Authors:** Miaomiao Liu, Shanfa Lu

**Affiliations:** Institute of Medicinal Plant Development, Chinese Academy of Medical Sciences and Peking Union Medical CollegeBeijing, China

**Keywords:** biosynthetic pathway, isoprenoid, metabolic engineering, plastoquinone, photosynthesis, respiration, secondary metabolism, ubiquinone

## Abstract

Plastoquinone (PQ) and ubiquinone (UQ) are two important prenylquinones, functioning as electron transporters in the electron transport chain of oxygenic photosynthesis and the aerobic respiratory chain, respectively, and play indispensable roles in plant growth and development through participating in the biosynthesis and metabolism of important chemical compounds, acting as antioxidants, being involved in plant response to stress, and regulating gene expression and cell signal transduction. UQ, particularly UQ_10_, has also been widely used in people’s life. It is effective in treating cardiovascular diseases, chronic gingivitis and periodontitis, and shows favorable impact on cancer treatment and human reproductive health. PQ and UQ are made up of an active benzoquinone ring attached to a polyisoprenoid side chain. Biosynthesis of PQ and UQ is very complicated with more than thirty five enzymes involved. Their synthetic pathways can be generally divided into two stages. The first stage leads to the biosynthesis of precursors of benzene quinone ring and prenyl side chain. The benzene quinone ring for UQ is synthesized from tyrosine or phenylalanine, whereas the ring for PQ is derived from tyrosine. The prenyl side chains of PQ and UQ are derived from glyceraldehyde 3-phosphate and pyruvate through the 2-*C*-methyl-D-erythritol 4-phosphate pathway and/or acetyl-CoA and acetoacetyl-CoA through the mevalonate pathway. The second stage includes the condensation of ring and side chain and subsequent modification. Homogentisate solanesyltransferase, 4-hydroxybenzoate polyprenyl diphosphate transferase and a series of benzene quinone ring modification enzymes are involved in this stage. PQ exists in plants, while UQ widely presents in plants, animals and microbes. Many enzymes and their encoding genes involved in PQ and UQ biosynthesis have been intensively studied recently. Metabolic engineering of UQ_10_ in plants, such as rice and tobacco, has also been tested. In this review, we summarize and discuss recent research progresses in the biosynthetic pathways of PQ and UQ and enzymes and their encoding genes involved in side chain elongation and in the second stage of PQ and UQ biosynthesis. Physiological functions of PQ and UQ played in plants as well as the practical application and metabolic engineering of PQ and UQ are also included.

## Introduction

**FIGURE 1:**
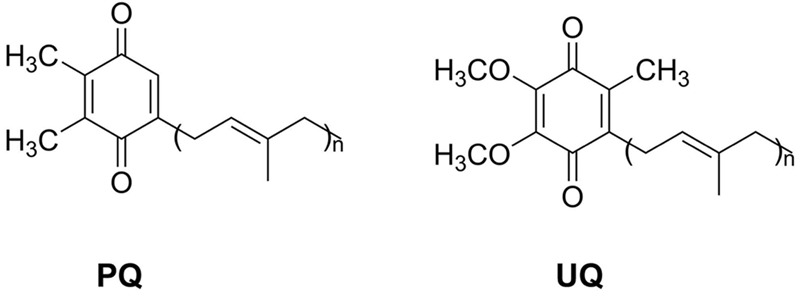
**The chemical structures of PQ and UQ.** PQ, plastoquinone; UQ, ubiquinone.

Plastoquinone (PQ) and ubiquinone (UQ) are two important prenylquinones functioning as electron transporters in plants. They are involved in photophosphorylation and oxidative phosphorylation located in chloroplast thylakoids and mitochondrial inner membrane, respectively (Swiezewska, 2004). PQ and UQ are both made up of an active benzoquinone ring attached to a polyisoprenoid side chain. The length of polyisoprenoid side chain determines the type of PQ and UQ. Difference between PQ and UQ in chemical structure mainly lies in diverse substituent groups of benzoquinone ring (**Figure 1**). Plant PQ and UQ usually include nine or ten units of isoprenoid side chain. For instance, the main PQ and UQ in *Arabidopsis thaliana* have nine such units, known as PQ_9_ and UQ_9_, respectively. PQ and UQ are localized in different organelles of plant cells. PQ is located on the thylakoids of chloroplasts, while UQ is located on the inner membrane of mitochondria. The locations of PQ and UQ are consistent with their roles in photophosphorylation and oxidative phosphorylation. Lifespan of PQ and UQ is very short in cells. The half-time of PQ and UQ in spinach cells is about 15 and 30 h, respectively (Wanke et al., 2000). Therefore, to maintain the concentration stable and dynamic balance for normal plant photosynthesis and respiration, PQ and UQ need to be continuously synthesized in cells. In addition to the significance of PQ and UQ in plants, UQ_10_ has also been used in people’s life. Significant progress has been made recently in PQ and UQ biosynthetic pathways and genes associated with PQ and UQ production. The biosynthesis and functions of UQ in *Escherichia coli, Saccharomyces cerevisiae* and animals (Clarke, 2000; Meganathan, 2001; Tran and Clarke, 2007; Bentinger et al., 2010; Aussel et al., 2014), UQ_10_ production in plants (Parmar et al., 2015) and metabolic engineering of UQ_10_ in microbes (de Dieu Ndikubwimana and Lee, 2014) have been reviewed. Here we mainly summarize and discuss recent advances in biosynthetic pathways, key enzymes and their encoding genes, physiological functions, and metabolic engineering of PQ and UQ in plants.

## Biosynthetic Pathways of PQ and UQ

**FIGURE 2:**
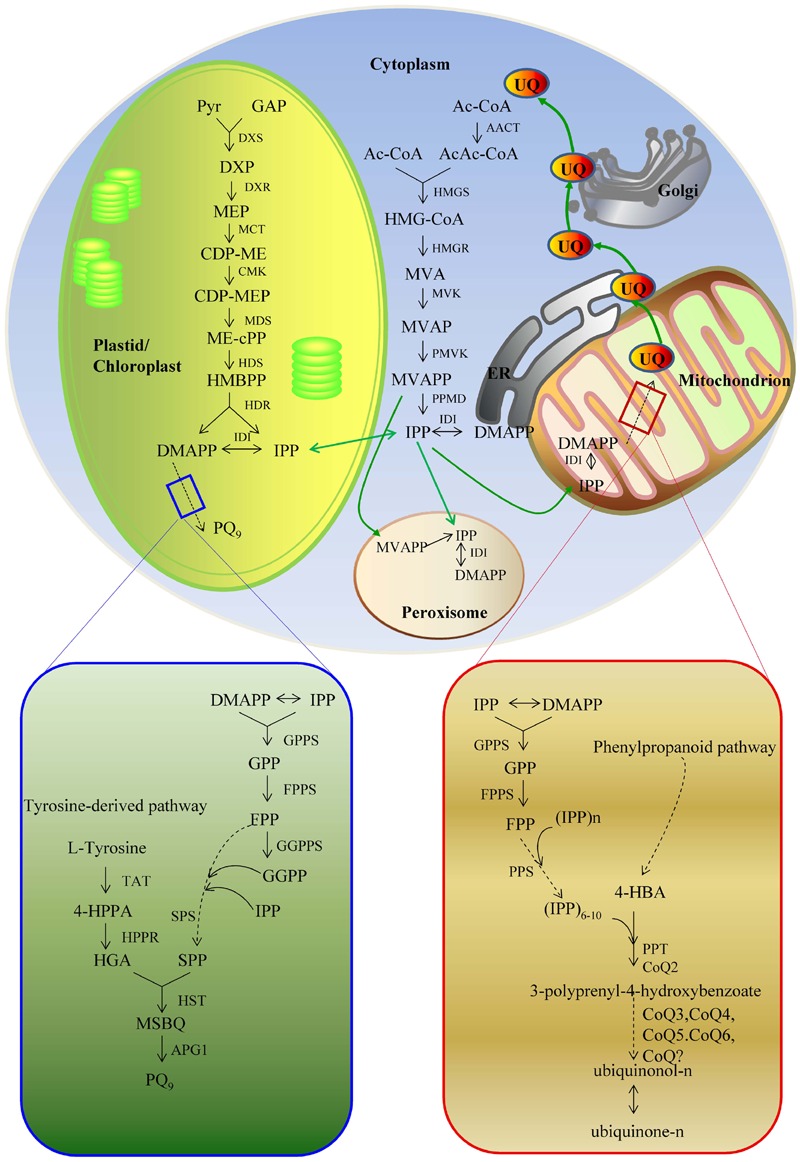
**The biosynthetic pathways of PQ_9_ and UQ.** 4-HBA, 4-hydroxybenzoate acid; 4-HPPA, 4-hydroxyphenylpyruvic acid; AACT, acetoacetyl-CoA thiolase; AcAc-CoA, acetyloacetyl-coenzyme A; Ac-CoA, acetyl-coenzyme A; APG1, MSBQ/MPBQ methyltransferase; CDP-ME, 4-diphosphocytidyl-2C-methyl-D-erythritol; CDP-MEP, 4-diphosphocytidyl-2C-methyl-D-erythritol 2-phosphate; CMK, 4-diphosphocytidyl-2-*C*-methyl-D-erythritol kinase; Coq?, unknown enzymes; Coq3 and Coq5, *O*-methyltransferase and *C*-methyltransferase; Coq6, monooxygenase; DMAPP, dimethylallyl pyrophosphate; DMAPP, dimethylallyl pyrophosphate; DPS, decaprenyl diphosphate synthese; DXP, 1-deoxy-D-xylulose-5 phosphate; DXR, 1-deoxy-D-xylulose 5-phosphate reductoisomerase; DXS, 1-deoxy-D-xylulose-5-phosphate; FPP, farnesyl diphosphate; FPPS, farnesyl diphosphate synthase; GAP, D-glyceraldehyde 3-phosphate; GGPP, geranylgeranyl diphosphate; GGPPS, geranylgeranyl diphosphate synthase; GPP, geranyl diphosphate; GPPS, geranyl diphosphate synthase; HDR, 1-hydroxy-2-methyl-2-(E)-butenyl 4-diphosphate reductase; HDS, 1-hydroxy-2-methyl-2-(E)-butenyl 4-diphosphate synthase; HGA, homogentisate acid; HMBPP, 1-hydroxy-2-methyl-2-(E)-butenyl 4-diphosphate; HMG-CoA, 3-hydroxy-3-methylglutaryl-coenzyme A; HMGR, 3-hydroxy-3-methylglutaryl-CoA reductase; HMGR, hydroxymethylglutaryl-CoA reductase; HMGS, 3-hydroxy-3-methylglutaryl-CoA synthase; HMGS, hydroxymethylglutaryl-CoA synthetase; HPPR, 4-hydroxyphenylpyruvate reductase; HST, homogentisate solanesyltransferase; IDI, isopentenyl-diphosphate *delta*-isomerase; IPP, isopentenyl diphosphate isomerase; IPP, isopentenyl diphosphate; MCT, 2C-methyl-D-erythritol 4-phosphate cytidyl transferase; MDS, 2C-methyl-D-erythritol 2,4-cyclodiphosphate synthase; ME-cPP, 2C-methyl-D-erythritol 2,4-cyclodiphosphate; MEP, 2C-methyl-D-erythritol-4-phosphate; MSBQ, methyl-solanesyl-benzoquinone; MVA, mevalonic acid; MVAP, mevalonate-5-phosphate; MVAPP, mevalonate-5-diphosphate; MVK, mevalonate kinase; PMVK, phosphomevalonate kinase; PPMD, diphosphomevalonate decarboxylase; PPS, diphosphate synthese; PPT/Coq2, 4-hydroxybenzoate polyprenyl transferase; Pyr, pyruvate; SPP, solanesyl diphosphate; SPS, solanesyl diphosphate synthase; TAT, tyrosine aminotransferase.

**FIGURE 3:**
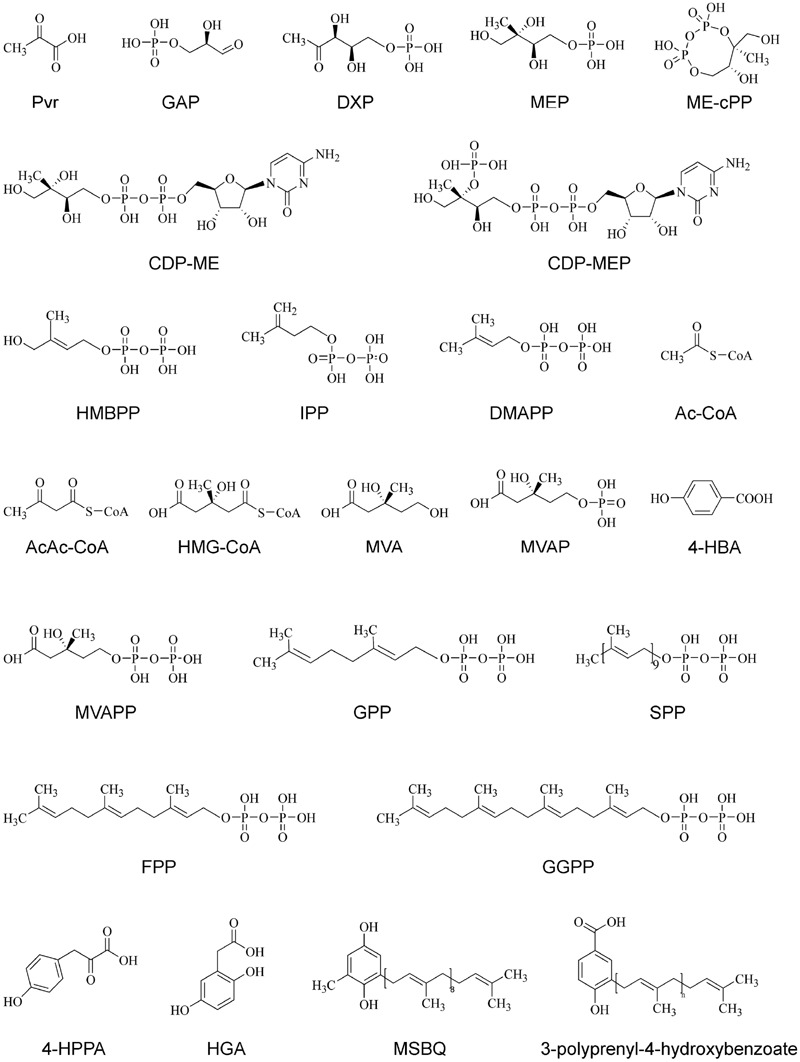
**The chemical structures of various compounds in PQ and UQ biosynthetic pathways.** 4-HBA, 4-hydroxybenzoate acid; 4-HPPA, 4-hydroxyphenylpyruvic acid; AcAc-CoA, acetyloacetyl-coenzyme A; Ac-CoA, acetyl-coenzyme A; CDP-ME, 4-diphosphocytidyl-2C-methyl-D-erythritol; CDP-MEP, 4-diphosphocytidyl-2C-methyl-D-erythritol 2-phosphate; DMAPP, dimethylallyl pyrophosphate; DMAPP, dimethylallyl pyrophosphate; DXP, 1-deoxy-D-xylulose-5 phosphate; DXS, 1-deoxy-D-xylulose-5-phosphate; FPP, farnesyl diphosphate; GAP, D-glyceraldehyde 3-phosphate; GGPP, geranylgeranyl diphosphate; GPP, geranyl diphosphate; HGA, homogentisate acid; HMBPP, 1-hydroxy-2-methyl-2-(E)-butenyl 4-diphosphate; HMG-CoA, 3-hydroxy-3-methylglutaryl-coenzyme A; IPP, isopentenyl diphosphate; ME-cPP, 2C-methyl-D-erythritol 2,4-cyclodiphosphate; MEP, 2C-methyl-D-erythritol-4-phosphate; MSBQ, methyl-solanesyl-benzoquinone; MVA, mevalonic acid; MVAP, mevalonate-5-phosphate; MVAPP, mevalonate-5-diphosphate; Pyr, pyruvate; SPP, solanesyl diphosphate.

The biosynthetic pathways of PQ and UQ can be generally divided into two stages (**Figures 2** and **3**). The first stage leads to the biosynthesis of precursors of benzene quinone ring and prenyl side chain. The second stage includes the condensation of ring and side chain and subsequent modification. The benzene quinone ring precursor for UQ is 4-hydroxybenzoic acid (4HB) synthesized from tyrosine or phenylalanine under the catalysis of various enzymes known as phenylalanine ammonia-lyase (PAL), cinnamic acid 4-hydroxylase (C4H), 4-coumarate CoA ligase (4CL), and other unknown enzymes. The benzene quinone ring precursor for PQ is homogentisic acid (HGA). It is synthesized from tyrosine under the catalysis of tyrosine aminotransferase (TAT) and 4-hydroxyphenylpyruvate reductase (HPPR). The prenyl side chains of PQ and UQ are derived from glyceraldehyde 3-phosphate (G3P) and pyruvate through the 2-*C*-methyl-D-erythritol 4-phosphate (MEP) pathway and/or acetyl-CoA and acetoacetyl-CoA through the mevalonate (MVA) pathway. The universal isoprene precursor isopentenyl diphosphate (IPP, C_5_) and its isomer dimethylallyl diphosphate (DMAPP) synthesized through the MEP and MVA pathways are converted into intermediate diphosphate precursors, including geranyl diphosphate (GPP, C_10_), farnesyl diphosphate (FPP, C_15_), and geranylgeranyl diphosphate (GGPP, C_20_). Enzymes catalyzing this conversion are a group of polyprenyl diphosphate synthases (PPSs), including geranyl diphosphate synthase (GPPS), farnesyl diphosphate synthase (FPPS), and geranylgeranyl diphosphate synthase (GGPPS) (Ma et al., 2012; Zhang and Lu, 2016). Both PQ and UQ share the prenyl side chain precursors. Side chain may be elongated to solanesyl diphosphate (SPP, C_45_) and decaprenyl diphospate (DPS, C_50_) under the catalysis of solanesyl diphosphate synthase (SPS) and decaprenyl diphosphate synthase (DPS), respectively. In the second stage, SPP is attached to HGA by homogentisate solanesyltransferase (HST) to produce intermediate 2-dimethyl-plastoquinone, which is then catalyzed by a methytransferase to form the end-product PQ_9_ in plants. The condensation of PHB and the corresponding prenyl side chain is catalyzed by PHB polyprenyltransferase (PPT). It leads to the formation of 3-polyprenyl-4-hydroxybenzoate. After three methylations, three hydroxylations and one decarboxylation, ubiquinonol-n and ubiquinone-n are formed eventually. Enzymes catalyzing these modification steps are currently not well understood.

## Enzymes and their Encoding Genes Involved in PQ and UQ Biosynthesis

Recently, genes involved in the MEP and MVA pathways have been intensively studied. Chemical compounds synthesized through these pathways are precursors for many terpenoids and their derivatives. They are not specific to PQ and UQ production. Here, we are not going to summarize the advances in these genes. Instead, genes involved in side chain elongation and in the second stage of PQ and UQ biosynthesis are reviewed in detail (**Table 1**).

**Table 1:** Genes involved in side chain elongation and in the second stage of PQ and UQ biosynthesis in photosynthetic organisms.

Gene	Enzyme	Organism	Function	Reference
*AtSPS1, AtSPS2*	Solanesyl diphosphate synthase	*Arabidopsis thaliana*	PQ_9_	Hirooka et al., 2003, 2005; Jun et al., 2004; Block et al., 2013; Ksas et al., 2015
*AtSPS3*	Solanesyl diphosphate synthase	*Arabidopsis thaliana*	UQ_9_	Hsieh et al., 2011; Ducluzeau et al., 2012; Block et al., 2013
*AtPPT1*/*HRL1*	4-hydroxybenzoate polyprenyltransferase	*Arabidopsis thaliana*	UQ_9_	Devadas et al., 2002; Okada et al., 2004; Dutta et al., 2015
*AtCoQ3*	*O*-methyltransferase	*Arabidopsis thaliana*	UQ_9_	Avelange-Macherel and Joyard, 1998
*AtCoQ5*	*C*-methyltransferase	*Arabidopsis thaliana*	UQ_9_	Hayashi et al., 2014
*AtCOQ6*	Monooxygenase	*Arabidopsis thaliana*	UQ	Hayashi et al., 2014
*AtHST*	Homogentisate solanesyltransferase	*Arabidopsis thaliana*	PQ_9_	Sadre et al., 2006; Tian et al., 2007; Chao et al., 2014
*APG1*/*VTE3*	MSBQ/MPBQ methyltransferase	*Arabidopsis thaliana*	PQ_9_ and tocopherol	Cheng et al., 2003; Motohashi et al., 2003; Naqvi et al., 2011
*BoCOQ5*	*C*-methyltransferase	*Brassica oleracea*	UQ_9_	Zhou et al., 2009
*BrCoQ3*	*O*-methyltransferase	*Brassica rapa*	UQ	Wang et al., 2011
*CrHST*	Homogentisate solanesyltransferase	*Chlamydomonas reinhardtii*	PQ_9_ and tocochromanol	Venkatesh et al., 2006
*CrAPG1*	MSBQ/MPBQ methyltransferase	*Chlamydomonas reinhardtii*	PQ and tocopherol	Cheng et al., 2003
*EsCoQ3*	*O*-methyltransferase	*Eutrema salsugineum*	UQ	Yang et al., 2013
*HbSDS*	Solanesyl diphosphate synthase	*Hevea brasiliensis*	PQ_9_	Phatthiya et al., 2007
*LsAPG1*	MSBQ/MPBQ methyltransferase	*Lactuca sativa*	tocopherol	Tang et al., 2016
*OsSPS1*	Solanesyl diphosphate synthase	*Oryza sativa*	UQ_9_	Ohara et al., 2010; Block et al., 2013
*OsSPS2, OsSPS3*	Solanesyl diphosphate synthase	*Oryza sativa*	PQ_9_	Ohara et al., 2010; Block et al., 2013
*OsPPT1a*	4-hydroxybenzoate Polyprenyltransferase	*Oryza sativa*	UQ_9_	Ohara et al., 2006
*SlSPS*	Solanesyl diphosphate synthase	Tomato	PQ_9_	Jones et al., 2013
*SlDPS*	Decaprenyl diphosphate synthase	Tomato	UQ_10_	Jones et al., 2013
*VTE3*	MPBQ methyltransferase	Peanut	methyltransferase	Liu et al., 2013
*APG1*	MSBQ/MPBQ methyltransferase	*Synechocystis* PCC6803	PQ and tocopherol	Shintani et al., 2002
*Slr0926*	4-hydroxybenzote solanesyltransferase	*Synechocystis* PCC6803	PQ_9_	Sadre et al., 2012

### Polyprenyl Diphosphate Synthase (PPS)

Polyprenyl diphosphate synthases, also known as prenyltransferases (PTs) and isoprenyl diphosphate synthases (IDSs), are a group of enzymes widely distributed in organisms. They catalyze the formation of polyprenyl diphosphates with various chain lengths through consecutive condensation of IPP and are key enzymes involved in the biosynthesis of isoprenoid compounds, including monoterpenes, diterpenes, triterpenoids, carotenoids, natural rubber and many derivatives, such as PQ and UQ, vatamin E, prenylflavonoids, and shikonnin (Kellogg and Poulter, 1997).

Polyprenyl diphosphate synthases possess seven common conserved domains, I–VII, of which domain II is characterized with the first aspartate-rich motif (FARM), DDX_2-4_D, while domain VI is characterized with the second aspartate-rich motif (SARM), DDXXD (Wang and Ohnuma, 1999; Phatthiya et al., 2007). These aspartate-rich motifs are involved in substrate binding and catalysis via chelating Mg^2+^, a cofactor required for enzyme activity (Wang and Ohnuma, 1999). Based on the chain length of final products, PPSs can be divided into three subfamilies: short- (C_15_–C_25_), medium- (C_30_–C_35_) and long-chain (C_40_–C_50_) PTs (Hemmi et al., 2002). The most common PQ and UQ in plants have a C_45_ or C_50_ prenyl side chain moiety. For instance, *Oryza sativa* PQ_9_ and UQ_9_ contain a C_45_ prenyl side chain (Ohara et al., 2010), while cauliflower and pea UQ_10_ has a C_50_ prenyl side chain (Mattila and Kumpulainen, 2001). Thus, long chain PPSs, such as SPS catalyzing the formation of solanesyl diphosphate (SPP, C_45_) and decaprenyl diphosphate synthase (DPS) involved in decaprenyl diphosphate (DPP, C_50_) production, are significant to PQ and UQ biosynthesis in plants.

Database mining of fully sequenced genomes showed that *Chlamydomnas reinhardtii, Cyanidioschyzon merolae, Cucumis sativus, Vitis vinifera* and *Hordeum vulgare* contained two *SPSs, Physcomitrella patens, Arabidopsis, Glycine max, Oryza sativa* and *Zea mays* had three, and *Brachypodium distachyon* contained four (Block et al., 2013). Among the three *Arabidopsis SPS* genes, *AtSPS1* (*At1g78510*) and *AtSPS2* (*At1g17050*) are highly expressed in leaves with the level of *AtSPS1* transcripts higher than *AtSPS2* (Hirooka et al., 2003, 2005), whereas *AtSPS3* (*At2g34630*) is ubiquitously expressed with peaks in seeds and shoot apical meristems (Ducluzeau et al., 2012). Although AtSPS1 had been shown previously to be localized in the ER (Hirooka et al., 2003, 2005; Jun et al., 2004), recent analysis have clearly demonstrated that both AtSPS1 and AtSPS2 is targeted exclusively to plastids and contribute to the biosynthesis of PQ_9_ (Block et al., 2013). Overexpression of *AtSPS1* resulted in the accumulation of PQ_9_ and its derivative plastochromanol-8 (PC_8_) (Ksas et al., 2015). Moreover, the enzymatic activity of AtSPS1 and AtSPS2 is stimulated by a member of the lipid-associated fibrillin protein family, fibrillin 5 (FNB5-B), which physically binds to the hydrophobic solanesyl moiety and helps to release the moiety from the enzymes in *Arabidopsis* cells (Kim et al., 2015). AtSPS3 had been shown previously to be targeted to plastids and to contribute to gibberellin biosynthesis (Bouvier et al., 2000). However, recent results suggest that it is actually dual targeted to mitochondria and plastids and appears very likely bifunctional (Ducluzeau et al., 2012). AtSPS3 is able to complement a yeast *coq1* knockout lacking mitochondrial hexaprenyl diphosphate synthase. Silence of *AtSPS3* using RNAi technology led to 75–80% reduction of the UQ pool size. *AtSPS3* overexpression resulted in a 40% increase in UQ content. No significant alternation of PQ levels was observed in *AtSPS3* silenced or overexpressing lines. Therefore, AtSPS3 seems to be the main contributor to SPS activity required for UQ_9_ biosynthesis in *Arabidopsis* (Hsieh et al., 2011; Ducluzeau et al., 2012).

Similarly, three *SPS* genes exist in *O. sativa* (Ohara et al., 2010; Block et al., 2013). *OsSPS1* is highly expressed in roots, whereas *OsSPS2* is highly expressed in both leaves and roots. TargetP prediction and transient expression of GFP fusion protein showed the localization of OsSPS1 in mitochondria and OsSPS2 in plastids. Recombinant proteins of both OsSPS1 and OsSPS2 catalyzed the formation of solanesyl diphosphates. The enzyme activity of OsSPS1 was stronger than OsSPS2. OsSPS1 complemented the yeast *coq1* disruptant and produced UQ_9_ in yeast cells, whereas OsSPS2 weakly complemented the growth defect of the *coq1* mutant (Ohara et al., 2010). The results suggest that OsSPS1 and OsSPS2 are involved in the supply of solanesyl diphosphate for UQ_9_ production in mitochondria and PQ_9_ biosynthesis in chloroplasts, respectively. *OsSPS3* is less studied compared with *OsSPS1* and *OsSPS2*. Since it shows high homology with OsSPS2 (Block et al., 2013), OsSPS3 appears to be also involved in PQ_9_ formation.

Analysis of the fully sequenced tomato genomes showed that *Solanum lycopersicum* contained two long-chain *PPSs* genes (Block et al., 2013). Jones et al. (2013) cloned and termed them *SlSPS* and *SlDPS*, respectively. *SlSPS* is targeted to plastids, whereas the fluorescence signal of SlDPS-GFP may resemble the mitochondrial localization reported for rice OsSPS1 (Ohara et al., 2010; Jones et al., 2013). In *E. coli*, SlSPS and SlDPS extend the side chain of endogenous UQ to nine and ten isoprene units, respectively (Jones et al., 2013). Overexpression of *SlSPS* elevated the content of PQ in immature tobacco leaves. Silence of *SlSPS* resulted in photobleached phenotype and accumulated phytoene. *SlSPS* and *SIDPS* could not complement silencing of each other. SlDPS can use GPP, FPP or GGPP in SPP and DPP biosynthesis. Silence of SlDPS did not affect leaf appearance, but impacted on primary metabolism (Jones et al., 2013). The roles of *SlSPS* and *SlDPS* in PQ and UQ biosynthesis need to be further investigated.

Although long chain PPSs play significant roles in PQ and UQ biosynthesis, the corresponding genes have only been identified from various plants, such as *Arabidopsis* (Hirooka et al., 2003, 2005; Jun et al., 2004; Ducluzeau et al., 2012; Block et al., 2013), rice (Ohara et al., 2010), tomato (Jones et al., 2013), and *Hevea brasiliensis* (Phatthiya et al., 2007). Greater efforts are required for molecular cloning and functional characterization of the genes in other plant species and utilization of plant source long chain *PPS*s in PQ and UQ production through biotechnology.

### Homogentisate Solanesyltransferase (HST)

HSTs catalyze the condensation of HGA and SPP to form 2-demethylplastoquinol-9 leading to PQ_9_ biosynthesis (Sadre et al., 2010). Although HSTs are members of the homogentisate PT family, they differ from other homogentisate PT members, such as the homogentisate phytyltransferases (HPTs) isolated from *Synechocystis* (slr1736) and *Arabidopsis* (VTE2), in enzyme properties (Collakova and DellaPenna, 2001; Savidge et al., 2002). HPTs showed the highest enzyme activities with phytyl diphosphate, whereas HSTs had the highest activities with solanesyl diphosphate and were hardly active with phytyl diphosphate (Sadre et al., 2006).

Compared with other genes involved in the upstream of PQ biosynthesis, HSTs are less studied, although they play an important role in the biosynthesis of PQ_9_, GAs and carotenoids, of which carotenoids are precursors of ABA and strigolactones. Genes encoding HSTs have only reported in *C. reinhardtii* and *Arabidopsis* (Sadre et al., 2006; Venkatesh et al., 2006; Tian et al., 2007; Sadre et al., 2010; Chao et al., 2014). The deduced *C. reinhardtii* CrHST and *Arabidopsis AtHST* proteins contain a chloroplast targeting sequence (Sadre et al., 2006; Tian et al., 2007). Confocal images of the AtHST-GFP fusion protein showed that AtHST protein was located in the chloroplast, most likely on the envelope membrane (Tian et al., 2007). Overexpression of *AtHST* caused modest elevation of PQ_9_ levels (Sadre et al., 2006). The *pds2* mutant with an in-frame 6 bp deletion in *AtHST* showed an albino phenotype and the mutation can be functionally complemented by constitutive expression of *AtHST* (Norris et al., 1995; Tian et al., 2007). Similarly, T-DNA insertion mutant of *HST* gene displayed albino, dwarf and early flowering phenotypes with chloroplast development arrested, chlorophyll (Chl) absent and stomata closure defected (Chao et al., 2014). The GA and ABA levels were very low in the mutant. Exogenous GA could partially rescue the dwarf phenotype and the root development defects. Exogenous ABA could rescue the stomata closure defects (Chao et al., 2014). The expression levels of many genes involved in flowering time regulation and PQ, Chl, GA, ABA and carotenoid biosynthesis were changed in the mutant, suggesting the key roles of *AtHST* played in chloroplast development and plant hormone biosynthesis (Chao et al., 2014). *HST* genes in other plant species remain to be isolated and characterized.

### 4-Hydroxybenzoate Polyprenyl Diphosphate Transferase (PPT)

PPT is a rate-limiting enzyme in UQ biosynthesis. It catalyzes the condensation of benzoquinone ring and polyisoprenoid side chain to form the intermediate, 3-polyprenyl-4-hydroxybenzoate. All of the reported eukaryotic PPTs contain a mitochondrial targeting signal at N-terminal sequence. Most PPTs have a broad specificity for prenyl diphosphates, accepting substrates of different chain lengths, but show a high specificity for 4HB (Forsgren et al., 2004; Okada et al., 2004; Ohara et al., 2006). *PPT* genes have been identified from various organisms, such as *E. coli* (Siebert et al., 1992), *S. cerevisiae* (Ashby et al., 1992), *Schizosaccharomyces pombe* (Uchida et al., 2000), human (Forsgren et al., 2004), and two plant species, including *Arabidopsis* (Okada et al., 2004) and rice (Ohara et al., 2006).

PPTs are members of the polyprenyl diphosphate transferase family. Although proteins in this family have sequence homology and relatively close phylogenetic relationship, only a small subset catalyzing the condensation of 4HB and prenyl chain are involved in UQ biosynthesis (Ohara et al., 2006). Other members may be involved in other metabolic pathways. For instance, *Cyanobacteria* hydroxybenzoate solanesyltransferase, Slr0926, which exhibits highly specific for 4-hybroxybenzoate and a broad specificity with regard to the prenyl donor substrate, including SPP and a number of shorter-chain prenyl diphosphates, is actually involved in PQ_9_ biosynthesis (Sadre et al., 2012). *Lithospermum erythrorhizon* 4HB geranyltransferases (*LePGT1* and *LePGT2*) with a strict specificity for GPP as a prenyl substrate are involved in shikonin biosynthesis and not relevant to UQ formation (Heide and Berger, 1989; Yazaki et al., 2002; Ohara et al., 2006, 2009).

*Arabidopsis AtPPT1* is the first *PPT* gene identified in plants (Okada et al., 2004). It is predominantly expressed in the flower cluster. The deduced AtPPT1 protein is localized in mitochondria and can complement the yeast *coq2* disruptant. AtPPT1 has broad substrate specificity in terms of the prenyl donor. The T-DNA insertion mutant of *AtPPT1* shows arrest of embryo development at an early stage of zygotic embryogenesis (Okada et al., 2004). The other mutant, known as *hypersensitive response-like lesions 1* (*hrl1*), was identified in an ethyl methanesulfonate (EMS)-mutagenesis screen (Devadas et al., 2002). The *hrl1* mutant spontaneously develops HR-like lesions and shows enhanced resistance against bacterial pathogens (Devadas et al., 2002). Positional cloning and subsequent DNA sequencing showed that the mutant had a single base change in an exon of *AtPPT1*. The mutation results in a leucine to phenylalanine substitution at position 228. Leucine 228 is not a part of the active site of the enzyme but is conserved across PPT sequences from various organisms (Dutta et al., 2015). Overexpression of *HRL1* in *A. thaliana* leads to elevated UQ and decreased ubiquinol levels (Dutta et al., 2015).

The *O. sativa* genome contains three *PPT* genes, including *OsPPT1a, OsPPT1b* and *OsPPT1c*. However, only OsPPT1a was found to be expressed (Ohara et al., 2006). The deduced OsPPT1a proteins contain a putative mitochondrial sorting signal at the N-terminus. Consistently, GFP-PPT fusion proteins are mainly localized in mitochondria (Ohara et al., 2006). Same as AtPPT1 and other PPT proteins, OsPPT1a can complement the yeast *coq2* mutant, accepts prenyl diphosphates of various chain lengths as prenyl donors, and shows strict substrate specificity for the aromatic substrate 4HB as a prenyl acceptor (Ohara et al., 2006).

### The Modification Enzyme of PQ Benzene Quinone Ring

2-demethylplastoquinol formed through the condensation of HGA and SPP by HSTs is a key intermediate in PQ synthesis. This intermediate can be converted to the final product PQ under the catalyzing of a modification enzyme, termed albino or pale green 1 (APG1) or methy-phytyl-benzoquinone (MPBQ)/methyl-solanesyl-benzoquinone (MSBQ) methyltransferase (Shintani et al., 2002; Motohashi et al., 2003; Cheng et al., 2003).

*Arabidopsis* APG1 was identified through characterization of the Ds-tagged albino or pale green mutant 1 (*apg1*) (Motohashi et al., 2003). This mutant lacks PQ, cannot survive beyond the seedling stage when germinated on soil, and contains decreased numbers of lamellae with reduced levels of chlorophyll (Motohashi et al., 2003). The insertion of Ds transposon disrupts a gene encoding a 37 kDa polypeptide precursor of the chloroplast inner envelope membrane. The 37 kDa protein had partial sequence similarity to the *S*-adenosylmethionine-dependent methyltransferase (Motohashi et al., 2003). Because of the lack of PQ in apg1 mutant and the putative methyltrasferase activity of the 37 kDa protein, APG1 appears to be involved in the methylation step of PQ biosynthesis (Motohashi et al., 2003). Almost at the same time, using a combined genomic, genetic and biochemical approach, Cheng et al. (2003) isolated and characterized the *Arabidopsis VTE3* (vitamin E defective) locus. This locus is actually identical to APG1. *In vitro* enzyme assays showed that *VTE3* was the plant functional equivalent of MPBQ/MSBQ methyltransferase from *Synechocystis* sp PCC6803 (Shintani et al., 2002).

In cyanobacterium, various genes involved in the PQ biosynthetic pathway and the vitamin E biosynthetic pathway are highly conserved. The biosynthesis of PQ and vitamin E share common precursor, HGA, which is prenylated with different substrates in the biosynthesis of different products. HGA is prenylated with phytyl diphosphate (PDP) or GGPP in tocochromanol formation, whereas it is prenylated with SPP in PQ_9_ biosynthesis. Thus, the MPBQ/MSBQ methyltransferase identified from *Synechocystis* sp PCC6803 has a dual function in the final methylation step of PQ and vitamin E biosynthesis (Shintani et al., 2002). Most genes involved in PQ and vitamin E synthesis are homologous, but MPBQ/MSBQ methyltransferase from *Synechocystis* sp PCC6803 and VTE3 from *Arabidopsis* are highly divergent in primary sequence (Cheng et al., 2003). The orthologs of *Synechocystis* MPBQ/MSBQ methyltransferase exist in *C. reinhardtii* and *Thalassiosira pseudonana* but absent from vascular and non-vascular plants. VTE3 orthologs exist in *C. reinhardtii* and vascular and non-vascular plants but absent from cyanobacteria (Cheng et al., 2003). It suggests that VTE3 is evolutionarily originated from archea rather than cyanobacteria. Interestingly, two mutants of *VTE3, vte3-1* and *vte3-2*, which show partial or total disruption of MPBQ/MSBQ methyltransferase activity, respectively, have different effects on PQ and vitamin E biosynthesis. *vte3-1* mainly impairs the methylation of tocopherol substrates. It has little effect on the methylation of MSBQ to PQ. On the contrary, *vte3-2* completely disrupts MPBQ/MSBQ methyltransferase (Shintani et al., 2002). The underlying mechanism remains to be elucidated.

Although genes encoding MPBQ/MSBQ methyltransferase are largely unknown in plant species other than *Arabidopsis*, engineering of *VTE3* has been tested (Van Eenennaam et al., 2003; Naqvi et al., 2011). Seed-specific expression of *Arabidopsis VTE3* in transgenic soybean reduced seed delta-tocopherol from 20 to 2%. Coexpression of *Arabidopsis VTE3* and *VTE4* (gamma-tocopherol methyltransferase gene) resulted in a greater than eightfold increase of alpha-tocopherol and an up to fivefold increase in vitamin E activity in transgenic soybean seeds (Van Eenennaam et al., 2003). Simultaneous expression of *Arabidopsis ρ*-hydroxyphenylpyruvate dioxygenase and *VTE3* in transgenic corn kernels triples the tocopherol content (Naqvi et al., 2011). It is currently unknown whether it is possible to increase the content of PQ in plants through *VTE3* overexpression.

### Modification Enzymes of UQ Benzene Quinone Ring

Compared with PQ benzene quinine ring modification, the process of UQ aromatic ring modification is complex. It includes three methylations (two *O*-methylations and one *C*-methylation), three hydroxylations, and one decarboxylation. UQ biosyntheses in prokaryotes and eukaryotes are similar. The difference lies in the reaction order of modifications. In eukaryotes, proposed modifications start with hydroxylation, followed by *O*-methylation, decarboxylation, two additional hydroxylations, *C*-methylation and one additional *O*-methylation (Tran and Clarke, 2007), whereas the reaction order of modifications in prokaryotes is decarboxylation, three hydroxylations, *O*-methylation, *C*-methylation and then an additional *O*-methylation (Meganathan, 2001).

#### Hydroxylation

The modification of UQ aromatic ring requires a total of three hydroxylations. The first hydroxylation of 3-polyprenyl-4-hydroxybenzoate in eukaryotes occurs before decarboxylation (Goewert et al., 1977). Two additional hydroxylations occur after decarboxylation in eukaryotes. Enzymes involved in hydroxylation are cytochrome P450 monooxygenases, known as COQ6 and COQ7 in yeast (Olson and Rudney, 1983). Genes encoding these enzymes have been identified from various organisms, such as *S. cerevisiae* (Marbois and Clarke, 1996; Kawamukai, 2000; Ozeir et al., 2011), rat (Jonassen et al., 1996), *Caenorhabditis elegans* (Ewbank et al., 1997), human (Vajo et al., 1999; Lu T.T. et al., 2013), and *E. coli* (Hajj Chehade et al., 2013). Homologs of the enzymes involved in UQ aromatic ring hydroxylations have also been found in various plants, such as *Arabidopsis* (Lange and Ghassemian, 2003) and alga (Blanc et al., 2010, 2012). *Arabidopsis* contains a COQ6 homolog, but lacks COQ7 (Hayashi et al., 2014). Although AtCOQ6 cannot complement the yeast *coq6* disruptant, addition of a mitochondrial targeting signal to AtCOQ6 will enable the production of UQ_10_ (Hayashi et al., 2014). Except AtCOQ6, other plant COQ6 and COQ7 homologs were identified based on genome sequence search and computational annotation, their roles in UQ biosynthesis need to be further confirmed using experimental approaches.

#### Methylation

The three methylations of UQ aromatic ring include two *O*-methylations and one *C*-methylation. The two *O*-methylation steps are catalyzed by COQ3, whereas the *C*-methylation step is catalyzed by COQ5 in yeast (Clarke et al., 1991; Barkovich et al., 1997; Poon et al., 1999; Hsu et al., 2000; Baba et al., 2004). COQ3 homologs have been identified in various organisms, such as *E. coli* (Hsu et al., 1996), yeast (Clarke et al., 1991; Poon et al., 1999; Hsu et al., 2000), rat (Marbois et al., 1994a,b) and human (Jonassen and Clarke, 2000). The gene encoding plant COQ3 was first identified in *Arabidopsis* (Avelange-Macherel and Joyard, 1998). *AtCOQ3* gene product is localized within mitochondrial membranes and can complement *Saccharomyces cerevisiae* or *Schizosaccharomyces pombe coq3* disruptant (Avelange-Macherel and Joyard, 1998; Hayashi et al., 2014). With the decoding of plant genomes, COQ3 has also been found in other plant species, such as *Brassica rapa* (Wang et al., 2011), *Eutrema salsugineum* (Yang et al., 2013). Similar to COQ3, COQ5 is associated with other COQ proteins on the inner mitochondrial membrane at the matrix side (Baba et al., 2004; Nguyen et al., 2014). *COQ5* genes have been identified in yeast (Barkovich et al., 1997; Baba et al., 2004), human (Chen et al., 2013; Nguyen et al., 2014), broccoli (Zhou et al., 2009) and *Arabidopsis* (Hayashi et al., 2014). *S. cerevisiae* COQ5 displays a typical class I *S*-adenosyl methionine-dependent methyltransferase crystal structure (Dai et al., 2014). The broccoli BoCOQ5 can complement a yeast *coq5* mutant and increases cellular UQ levels in bacteria (Zhou et al., 2009). Similarly, AtCOQ5 can complement the *Schizosaccharomyces pombe coq5* disruptant (Hayashi et al., 2014).

#### Decarboxylation

The proposed modifications of UQ aromatic ring include a decarboxylation step. It occurs before the three hydroxylation steps in prokaryotes, whereas in eukaryotes, decarboxylation occurs after a hydroxylation step and an *O*-methylation step. It has been shown that *ubiX* and *ubiD* genes are involved in the decarboxylation of UQ aromatic ring in bacteria (Meganathan, 2001; Gulmezian et al., 2007; Aussel et al., 2014). *ubiX* encodes a flavin mononucleotide (FMN)-binding protein with no decarboxylase activity detected *in vitro* (Gulmezian et al., 2007; Aussel et al., 2014), and UbiX proteins are metal-independent and require dimethylallyl-monophosphate as substrate (White et al., 2015). During the biosynthesis of UQ, UbiX acts as a flavin PT, producing a flavin-derived cofactor required for the decarboxylase activity of UbiD (Payne et al., 2015; White et al., 2015). *pad1* and *fdc1* are two fungal genes related to bacterial *ubiX* and *ubiD* (Mukai et al., 2010; Lin et al., 2015; Payne et al., 2015). Although Pad1 and Fdc1 proteins are homologous with UbiX and UbiD, respectively, and UbiX can activate Fdc1, they are not essential for UQ synthesis in yeast (Mukai et al., 2010; Lin et al., 2015). Thus, the decarboxylation step is largely unknown in eukaryotes. Feeding the isolated mitochondria from potato tubers with ^14^C-labeled IPP, 4HB and *S*-adenosylmethionine shows the accumulation of methoxy-4-hydroxy-5-decaprenylbenzoate. It indicates the occurrence of decarboxylation after the first hydroxylation and subsequent *O-*methylation in plants; however, the immediate methoxy-4-hydroxy-5-decaprenylbenzoate cannot be detected in potato tubers (Lütke-Brinkhaus et al., 1984; Lütke-Brinkhaus and Kleinig, 1987), and no plant genes and enzymes involved in the decarboxylation of UQ aromatic ring have been identified.

In addition to the enzymes mentioned above, various other enzymes, such as yeast COQ4, COQ8, and COQ9, are potentially involved in UQ benzene quinone ring modification, although their actual functions are currently unknown (Tran and Clarke, 2007). Moreover, it seems that modification enzymes involved in modification of UQ benzene quinone ring form a multi-subunit complex. The interaction among subunits guarantees the normal function of enzymes.

## Physiological Functions of PQ and UQ in Plants

Both PQ and UQ are functionally important electron transporters in plants. PQ is involved in the electron transport chain of oxygenic photosynthesis, whereas UQ works exclusively as an electron carrier in the aerobic respiratory chain (Cramer et al., 2011; de Dieu Ndikubwimana and Lee, 2014; Parmar et al., 2015). In addition to their basal functions in photophosphorylation and oxidative phosphorylation, PQ and UQ also play indispensable roles in plant growth and development through participating in the biosynthesis or metabolism of various important chemical compounds, acting as antioxidants, being involved in plant response to stress, and regulating gene expression and cell signal transduction.

### Involved in Biosynthesis or Catabolism of Chemical Compounds

It has been shown that PQ and UQ are involved in the biosynthesis or metabolism of various important chemical compounds in plants. For instance, PQ participates in the biosynthesis of carotenoids (Norris et al., 1995), abscisic acid (ABA) (Rock and Zeevaart, 1991) and gibberellin (GA) (Nievelstein et al., 1995), whereas UQ is involved in branch-chain amino acid metabolism (Ishizaki et al., 2006; Araújo et al., 2010).

Carotenoids are C_40_ tetraterpenoids functioned as accessory light-harvesting pigments in photosynthetic tissues. In non-photosynthetic tissues, such as fruits and flowers, high levels of carotenoids often bring intense orange, yellow and red colors (Pfander, 1992). During carotenoid biosynthesis, the phytoene desaturation reaction is a rate-limiting step. A certain quinone/hydroquinone balance is necessary for optimal phytoene desaturation (Mayer et al., 1990). In an anaerobic environment, the oxidized quinones rather than reduced quinones are involved in the desaturation of phytoene (Mayer et al., 1990). *pds1* and *pds2* are two *Arabidopsis* mutants showing albino phenotype (Norris et al., 1995). The mutations affect phytoene desaturation and cause accumulation of phytoene, but they are not occurred in the phytoene desaturation enzyme. Analysis of *pds1* and *pds2* shows that *pds1* is 4-hydroxypheylpyruvate dioxygenase deficient (Norris et al., 1998), whereas *pds2* is deficient in HST, a critical enzyme involved in PQ biosynthesis (Tian et al., 2007). Both of the mutations lead to plastoquionone/tocopherol absence from different aspects in *Arabidopsis*, providing conclusive evidence that PQ is an essential component in phytoene desaturation (Nievelstein et al., 1995; Norris et al., 1995).

Since the plant hormone ABA is synthesized by oxidative cleavage of epoxy-carotenoids (Rock and Zeevaart, 1991), it is reasonable that PQ is also important for ABA biosynthesis. In a T-DNA insertion mutant of *HST* gene (*pds2-1*), not only PQ but also carotenoids, ABA and GA_3_ levels are dramatically reduced (Chao et al., 2014). PQ works as the co-factor of phytoene desaturase and ζ-carotene desaturase and is the immediate electron acceptor in carotenoid and ABA biosynthesis (Chao et al., 2014). Disruption of *HST* gene results in PQ content decrease, which subsequently affects carotenoid and ABA biosynthesis. On the other hand, the biosynthesis of PQ, carotenoid, ABA and GA shares the common precursor, GGPP (Ma et al., 2012; Chao et al., 2014; Du et al., 2015; Zhang and Lu, 2016). In the *pds2-1* mutant, expression of GA biosynthesis genes, such as *GA1, GA2*, and *GA3*, is significantly down-regulated (Chao et al., 2014). Consistently, in the *Arabidopsis* phytoene desaturase gene mutant (*pds3*), gibberellin biosynthesis is impaired (Qin et al., 2007). It indicates that PQ may affect the biosynthesis of other chemical compounds through negative feedback regulation or other indirect mechanisms.

The relationship between UQ and amino acids lies in two aspects: (1) The precursors of UQ biosynthesis are derived from amino acids, including phenylalanine and tyrosine; and (2) UQ is involved in catabolism of some branched-chain amino acids in mitochondria. It is well known that mitochondrion is an integration point of cellular metabolism and signaling. Amino acids are not only metabolized in peroxisomes but also broken down in mitochondria, which provide carbon skeletons for biosynthesis of many important compounds, such as vitamins, amino acids, and lipids (Sweetlove et al., 2007). In *Arabidopsis*, leucine is catabolized to form isovaleryl-CoA in mitochondrial matrix, and then the intermediate of the leucine catabolic pathway, isovaleryl-CoA, is dehydrogenated to 3-methylcrotonyl-CoA by isovaleryl-CoA dehydrogenase (IVD). This process occurs on the matrix face of the inner mitochondrial membrane and an electron is transferred through the electron-transfer flavoprotein/electron-transfer falvoprotein:ubiquinone oxidoreductase (ETF/ETFQO) system, first to flavoprotein and then to flavoprotein:ubiquione oxidoreductase (Ishizaki et al., 2006; Araújo et al., 2010). UQ is the final acceptor of electrons in the decomposition of leucine. Similarly, electrons produced during the catabolism of lysine can also be channeled to the mitochondrial electron transport chain (Araújo et al., 2010).

### Act as Antioxidants and Involved in Plant Response to Stress

Plastoquinone and ubiquinone can scavenge free radicals to prevent lipid peroxidation, protein oxidation and DNA damage in plant response to biotic and abiotic stresses. They exert antioxidant activity in the reduced forms, plastoquinol and uibiquinol, located in chloroplast thylakoid membrane and mitochondrial membrane, respectively. Analysis of the antioxidant effect of reduced PQ in isolated spinach thylakoid membranes showed that the reduced PQ acted as a scavenger of toxic oxygen species generated in the thylakoid membranes under strong illumination stress (Hundal et al., 1995). Reduced PQ inhibits lipid peroxidation and pigment bleaching, whereas oxidized PQ plays an opposite role (Hundal et al., 1995). In PQ-depleted spinach PSII membranes, exogenously added plastoquinol serves as an efficient scavenger of singlet oxygen (Yadav et al., 2010). Similarly, in *C. reinhardtii* cells, the level of reduced PQ markedly increased under high-light stress. When pyrazolate, an inhibitor of PQ and tocopherol biosynthesis, was added, the content of reduced PQ quickly decreases (Kruk et al., 2005). Further analyzing the turnover of plastoquinol showed that, due to scavenging of singlet oxygen, the reduced PQ underwent high turnover rate under high-light conditions (Kruk and Trebst, 2008). Moreover, the redox state of PQ pool was found to be an upstream master switch associated with programmed cell death in *Arabidopsis* leaves in response to excess excitation energy and may be play a central role in the light acclimation of diatoms (Mühlenbock et al., 2008; Lepetit et al., 2013). PSII photoinhibition occurred as a consequence of more reduced PQ pool (Darwish et al., 2015).

In addition to abiotic stress, PQ is also involved in plant response to biotic stress. When *Solanum nigrum* was treated with the pathogen *Phytophthora infestans*-derived elicitor, reactive oxygen species (ROS) production, lipid peroxidation and lipoxygenase activity were elevated (Maciejewska et al., 2002). These events were accompanied by a significant increase in PQ level. The increase of PQ level was more significant in plants growing in darkness than under continuous light. It suggests that PQ may be involved in maintaining a tightly controlled balance between the accumulation of ROS and antioxidant activity (Maciejewska et al., 2002). *Mesembryanthemum crystalinum* performs C_3_ and CAM carbon metabolism. Analysis of *M. crystalinum* plants infected with pathogen *Botrytis cinere* showed that the redox state of PQ pool modifies plant response to biotic stress and hypersensitive-like response is accelerated when PQ pool is in the reduced state (Nosek et al., 2015).

Ubiquinone is an obligatory element of mitochondrial functions in both animals and plants. The antioxidant activity of UQ has been extensively characterized in animals (Littarru and Tiano, 2007, 2010). It prevents DNA damage and cell membrane lipid peroxidation through the elimination of ROS. Same as PQ, UQ also has two forms: the reduced type (ubiquinol) and the oxidized one (ubiquinone), of which ubiquinol is the form exerting antioxidant activity. Overexpression of yeast *coq2* (*p*-hydroxybenzoate poliprenyltransferase) in tobacco resulted in the increase of UQ in transgenic lines (Ohara et al., 2004). Transgenics with the higher UQ level showed the greater tolerance to oxidative stresses caused by methyl viologen or high salinity (Ohara et al., 2004). Analysis of the suspension-cultured *Chorispora bungeana* cells showed that the redox transition of UQ played key roles in adaptation of cellular regulations under chilling stress (Chang et al., 2006). In addition, the redox state of UQ determines the levels of ROS and plays a key regulatory role in *Arabidopsis* basal resistance against bacterial pathogens and in response to high oxidative stress environments (Dutta et al., 2015).

Actually, the PQ and UQ pools play a dual role: (1) reducing O_2_ to superoxide by semiquinone; and (2) reducing superoxide to hydrogen peroxide by hydroquinone. In plant cells, the predominant ROS involved in plant defense includes superoxide and hydrogen peroxide, which are distributed in different pools. Moreover, ROS are generated in two ways. One is elicited by external stresses, such as environmental stresses and biotic stresses. The other way is produced through metabolic processes in the cells, such as the electron transport chains in mitochondria and chloroplasts (Mubarakshina and Ivanov, 2010; Tripathy and Oelmuller, 2012). An unknown interaction may be existed between different pools in modulation of ROS generation and plant response to stress.

### Regulate Cell Signal Transduction and Gene Expression

Plastoquinone and ubiquinone may regulate cell signaling and gene expression indirectly through the generation of hydrogen peroxide, an important signaling molecule in plant resistance and cell metabolism. They can also directly regulate the expression of genes involved in cell metabolism. For instance, UQ_10_ influences the expression of hundreds of human genes involved in different cellular pathways. Among them, seventeen are functionally connected by signaling pathways of G-protein coupled receptor, JAK/STAT, integrin and β-arrestin (Schmelzer et al., 2007). These UQ_10_-inducible genes possess a common promoter framework with binding domains of transcription factor families EVII, HOXF, HOXC and CLOX (Schmelzer et al., 2007). Moreover, UQ_10_–mediated gene-gene network are involved in inflammation, cell differentiation, and peroxisome proliferator-activated receptor signaling (Schmelzer et al., 2011).

In plants, Chla/b-binding protein complex II (LHC II) and NADPH dehydrogenase complex are two important protein complexes in photosynthesis. The cytochrome b_6_f deficient mutant of *lemna perpusilla* maintains a low level of the light-harvesting chl a/b-binding protein complex II (LHC II) at low-light intensities (Yang et al., 2001). Inhibiting the reduction of PQ pool increases the level of LHC II in the mutant at both low- and high-light intensities, whereas the level of LHC II is increased in wild-type plants only under high-light conditions (Yang et al., 2001). It suggests that the redox state of PQ is an important signal-transducing component in plant photoacclimation process (Yang et al., 2001). Analysis of gene expression using DNA microarray technology showed that 663 genes were differentially expressed in *A. thaliana* under low, medium, high and excessive irradiances, of which 50 genes were reverted by 3-(3,4-dichlorophenyl)-1,1-dimethylurea (DCMU), an inhibitor of the photosynthetic electron transport chain (Adamiec et al., 2008). It indicates that the expression of the 50 genes is regulated by the redox state of PQ pool. Hierarchical clustering and promoter motif analysis showed that the promoter regions of PQ-regulated genes contain conserved *cis*-acting elements involved in signal transduction from the redox state of the PQ pool (Adamiec et al., 2008). Active NADPH dehydrogenase complex is necessary for cyclic electron transport in photosystem I (PSI) and respiration. In glucose-treated cyanobacteria *Synechocystis* sp. strain PCC 6803 cells, NADPH dehydrogenase complex activity was inhibited and the cyclic PSI rate was decreased. In contrast, when the cells were treated with DCMU, the activity of NADPH dehydrogenase was significantly stimulated (Ma et al., 2008). Glucose treatment causes partial reduction of the PQ pool, whereas DCMU results in overoxidation. Differential responses of enzyme activity and cyclic PSI rate to glucose and DCMU treatments indicate that the redox state of PQ pool controls the NADPH dehydrogenase complex activity and further influences on cyclic PSI (Ma et al., 2008).

In addition to PQ, UQ is also involved in signal transduction in plants. Comparative analysis of hypersensitive tobacco *Nicotiana tabacum L.* variety *Samsun NN* treated with UQ_10_ and TMV and those treated with TMV only showed that UQ_10_ and TMV-treated tobbaco had less number of lesions and TMV and greater change of plant hormone levels, including the decrease of ABA and increase of IAA level (Rozhnova and Gerashchenkov, 2006). It indicates that UQ_10_ has a protective antiviral effect through controlling plant hormonal status (Rozhnova and Gerashchenkov, 2008). On the other hand, it has been reported that the ROS level generated by UQ redox state is a threshold for successful basal resistance response in plants (Dutta et al., 2015). In plant defenses, ROS acts as signaling molecules directly or mediates the generation of phytoalexins or serves as a source for activation of further defenses indirectly (Kovtun et al., 2000; Thoma et al., 2003; Mur et al., 2008). Plants employ both pathogen-associated molecular pattern (PAMP)-triggered immunity (PTI) and effector-triggered immunity (ETI) in basal and R gene-mediated defense response. ROS is induced rapidly and transiently and then mediates signaling during PTI and ETI (Frederickson Matika and Loake, 2013). The induction of ROS is considered as a defining hallmark of identification and subsequent defense activation against pathogens (Torres et al., 2006; Dutta et al., 2015). Moreover, UQ is involved in the mitochondrial glycerol-3-phosphate shuttle for redox homeostasis in plants (Shen et al., 2007) and serves as mitochondrial permeability transition pore in cell metabolism (Amirsadeghi et al., 2007; Reape et al., 2007).

## Utilization and Metabolic Engineering of PQ and UQ

### Utilization of PQ and UQ

Plastoquinone is specific to plants. It has not been directly utilized for human. However, various synthesized PQ derivatives, such as SkQ1 (plastoquinonyl-decyl-triphenylphosphonium), SkQR1 (the rhodamine- containing analog of SkQ1) and SkQ3 (methylplastoquinonyl-decyl-triphenylphosphonium), were reported to show antioxidant and protonophore activity (Skulachev et al., 2011). They are able to penetrate cell membranes and potentially used in anti-aging treatment (Anisimov et al., 2008, 2011; Obukhova et al., 2009). SkQ1 is currently under clinical trials for glaucoma treatment in Russia (Iomdina et al., 2015). The phase 2 clinical trial indicates that SkQ1 is safe and efficacious in treating dry eye signs and symptoms (Petrov et al., 2016). In plants, SkQ1 and SkQ3 can retard the senescence of *Arabidopsis* rosette leaves and their death, increase the vegetative period, and improve crop structure of wheat (Dzyubinskaya et al., 2013). In addition, SkQ1 effectively suppresses the development of p50-induced PCD in tobacco plants through inhibiting ROS production (Solovieva et al., 2013; Samuilov and Kiselevsky, 2015). The role of SkQ1 and SkQ3 played in cells is mainly based on its antioxidant activity.

Compared with PQ, the practical application of UQ, particularly UQ_10_, has attracted more attention. UQ_10_ is effective in treating cardiovascular diseases, particularly in preventing and treating hypertension, hyperlipidemia, coronary artery disease and heart failure (Tran et al., 2001; Moludi et al., 2015). In the past decades, many studies have reported the remarkable clinical benefits of UQ_10_ and illuminated its antioxidant activity as the basis of pathology (Lee et al., 2012). Moreover, UQ_10_ controls energy metabolism and regulates cell death via redox signaling, indicating its potential in cancer treatment (Chai et al., 2010). Moderate UQ_10_ levels have favorable impact on breast cancer (Folkers et al., 1993; Lockwood et al., 1994; Premkumar et al., 2008). In addition, UQ_10_ can enhance viral immunity and affect the development of AIDS (Folkers et al., 1988, 1991). UQ_10_ is also closely related to human reproductive health. Moderate UQ_10_ supplement can effectively reduce the risk of spontaneous abortion and develop pre-eclampsia in pregnant women (Palan et al., 2004; Teran et al., 2009). Exogenous administration of UQ_10_ can increase sperm cell motility and the mean pregnancy rate. Its positive role in the treatment of male infertility also relies on the antioxidant properties and bioenergetics (Balercia et al., 2004; Mancini et al., 2005; Balercia et al., 2009; Mancini and Balercia, 2011; Safarinejad, 2012). As a good immunomdulator, UQ_10_ has been used to treat chronic gingivitis and periodontitis (Chatterjee et al., 2012; Hans et al., 2012). A series of toothpaste containing UQ_10_ are developed and sold in the market. Other UQ_10_-containing chemical products include cleanser, cosmetic products, and healthy foods. Similar to PQ derivatives, the role of UQ played in cells relied on the antioxidant activity. As an antioxidant, UQ effectively scavenge ROS and prevent ROS-induced damage to membrane lipids, DNA, and proteins.

### Metabolic Engineering of UQ

Plastoquinone plays significant roles in plants; however, it is not directly used in human life. Moreover, PQ derivatives are mainly synthesized by chemical methods. Metabolic engineering of PQ and its derivatives is rarely reported. Differing from PQ, metabolic engineering of UQ, particularly UQ_10_, has been conducted in prokaryotes and eukaryotes, including bacterial, yeast and plants. Currently, the majority of commercially available UQ_10_ comes from yeast fermentation and chemical synthesis. Compared with microbial fermentation, chemical synthesis of UQ_10_ is more expensive and produces environmentally harmful waste products. Additionally, the scalability of both yeast fermentation and chemical synthesis is limited. Thus, plants are thought to be an attractive alternative source of UQ_10_.

The natural UQ producers, such as *Agrobacterium tumefaciens, Paracoccus denitrificans, Rhodobacter sphaeroides*, and their chemical mutants have been successfully used for commercial production of UQ; while, with the increase of knowledge about enzymes involved in UQ biosynthesis and regulatory mechanisms modulating UQ production, opportunities have arisen for UQ metabolic engineering in other organisms. For instance, overexpression of some key genes, such as *ubiA* encoding *p*-hydroxybenzoate-polyprenyl pyrophosphate transferase, *ispB* encoding polyprenyl pyrophosphate synthetase and *ubiCA*, in *E. coli* may achieve the level of UQ content 3-4 times to that of wild-type cells (Zhu et al., 1995; Jiang et al., 2006). Even though, UQ production using these methods does not meet industrial needs, which require a yield of higher than 500 mg/L (Cluis et al., 2007). Therefore, in addition to the highly efficient microbial system, growth condition optimization and alteration of cellular regulatory mechanisms are important for UQ production (Sakato et al., 1992; Zhang et al., 2007a,b). Recently, multiple strategies have been employed in improving UQ production. One hundred and eighty percent increase of UQ_8_ content is achieved in *E. coli* (*ΔmenA*) through a comprehensive approach, including blocking menaquinone pathway, coexpressing *dxs-ubiA*, and supplementing PYR and pHBA (Xu et al., 2014). The highest UQ10 titer and yield, 433 mg/L, is obtained in engineered *E. coli* through integrating *dps* into chromosome of *E. coli* ATCC8739, modulating *dxs* and *idi* genes of the MEP pathway and *ubiCA* genes, and recruiting the glucose facilitator protein of *Zymomonas mobilis* was to replace native phosphoenolpyruvate: carbohydrate phosphotransferase systems (PTS) (Dai et al., 2015).

Metabolic engineering of UQ in plants mainly concentrated on UQ_10_ production. Although UQ widely exists in plant cells, most cereal crops produce mainly UQ_9_. Tomato, *Datura tatula* and tobacco BY-2 cells can produce UQ_10_ naturally; however, its yield is very limited (Ikeda and Kagei, 1979; Ikeda et al., 1981; Matsumoto et al., 1981). It has been shown that overexpression of rate-limiting genes and increase of UQ precursors can improve UQ production in plants. Expression of *ddsA* from *Gluconobacter suboxydans* in rice leads to efficient production of UQ_10_ in rice seeds (Sakiko et al., 2006, 2010). Since PPT plays the catalyzing role in a rate-limiting step of the UQ biosynthesis pathway, it is a significant target for UQ metabolic engineering in plants (Ohara et al., 2004; Stiff, 2010; Parmar et al., 2015). For instance, expression of yeast *coq2* gene resulted in a sixfold increase of UQ_10_ levels in transgenic tobacco plants (Ohara et al., 2004). Compared with wild type plants, *coq2* transgenic tobacco with high UQ_10_ level are more resistant to oxidative stresses caused by methyl viologen or high salinity (Ohara et al., 2004). Similarly, overexpression of *AtPPT1* in tobacco increases UQ_10_ content and enhances oxidative stress tolerance caused by high NaCl (Stiff, 2010). Increase of UQ precursors, such as 4HB and/or PPS, may potentially improve UQ production in plants (Sommer and Heide, 1998; Viitanen et al., 2004). However, due to the complex relationship among precursors, UQ production and many other intersecting metabolic pathways, the expected goal of improving UQ production is difficult to achieve. Further improvement of UQ content in plant cells may be expected using comprehensive approaches (Kumar et al., 2012), such as improving the amount of UQ precursors combined with overexpression of rate-limiting genes.

## Conclusion and Perspectives

Plastoquinone and ubiquinone are two important compounds in plants. They function as electron transporters in the electron transport chain of oxygenic photosynthesis and the aerobic respiratory chain, respectively, and play indispensable roles in plant growth and development. UQ, particularly UQ_10_, has also been widely used in people’s life. Great efforts have been done to elucidate their biosynthetic pathways and genes associated with PQ and UQ production. As shown in **Figure 2** and **Table 1**, significant achievements have been made. However, there are still several issues related to the biosynthetic pathways, regulatory mechanisms and metabolic engineering need to be addressed.

Although a great amount of studies have been done to the MEP and MVA pathways and various enzymes, such as PPT and HST, involved in the attachment of isoprenoid side chain to the benzoquinone ring, pathways and enzymes involved in isoprenoid side chain elongation, UQ benzoquinone ring biosynthesis, and PQ and UQ benoquinone ring modification are largely unknown. The key enzyme responsible for 4HB production has not been identified (Block et al., 2014). PPSs are a group of enzymes converting IPP and DMAPP to diphosphate precursors. Each PPS may play different role in isoprenoid side chain elongation, which needs to be clarified. Although various enzymes involved in benoqunone ring modifications have been identified in bacteria and yeast, few achievements have been made in plants. With more and more transcriptome and whole genome sequence available, gene network reconstruction becomes possible and can be used to address these problems.

Non-coding RNAs, including small RNAs and long non-coding RNAs, play significant regulatory roles in many aspects of plants (Lu et al., 2005, 2007, 2008; Lu S. et al., 2013; Li D. et al., 2015; Wang et al., 2015). Various microRNAs have been identified to be associated with secondary metabolism (Wu et al., 2012; Lu S. et al., 2013; Fan et al., 2015; Wei et al., 2015). However, the regulatory roles of non-coding RNAs in PQ and UQ biosynthesis have not been revealed. In addition to non-coding RNAs, transcription factors, such as MYB, WRKY and SPL, potentially play significant regulatory roles in PQ and UQ biosynthesis (Li and Lu, 2014a,b; Zhang et al., 2014; Li C. et al., 2015), which needs to be further demonstrated. Understanding the regulatory mechanisms of PQ and UQ are important for manipulating the content of PQ and UQ in plants.

Metabolic engineering of UQ_10_ has been successfully performed in bacteria and yeast. However, they were found to be low yield and high production cost. UQ_10_ metabolic engineering in plants has various advantages and great perspectives, whereas current efforts are limited to a few plant species (Parmar et al., 2015). Increasing UQ_10_ content in UQ_10_-producing plant species and engineering UQ_10_ in non-UQ_10_-producing plant species are two routes for UQ_10_ metabolic engineering in plants. With more and more genes involved in UQ_10_ biosynthesis and regulation to be identified, great achievements may be expected for UQ_10_ production in plants.

## Author Contributions

Both authors listed, have made substantial, direct and intellectual contribution to the work, and approved it for publication.

## Conflict of Interest Statement

The authors declare that the research was conducted in the absence of any commercial or financial relationships that could be construed as a potential conflict of interest.
